# Divergent SARS CoV-2 Omicron-reactive T- and B cell responses in COVID-19 vaccine recipients

**DOI:** 10.1126/sciimmunol.abo2202

**Published:** 2022-02-03

**Authors:** Corine H. GeurtsvanKessel, Daryl Geers, Katharina S. Schmitz, Anna Z. Mykytyn, Mart M Lamers, Susanne Bogers, Sandra Scherbeijn, Lennert Gommers, Roos S.G. Sablerolles, Nella N. Nieuwkoop, Laurine C. Rijsbergen, Laura L.A. van Dijk, Janet de Wilde, Kimberley Alblas, Tim I. Breugem, Bart J.A. Rijnders, Herbert de Jager, Daniela Weiskopf, P. Hugo M. van der Kuy, Alessandro Sette, Marion P.G. Koopmans, Alba Grifoni, Bart L. Haagmans, Rory D. de Vries

**Affiliations:** ^1^ Department of Viroscience, Erasmus MC, Rotterdam, the Netherlands; ^2^ Department of Hospital Pharmacy, Erasmus MC, Rotterdam, Netherlands; ^3^ Department of Medical Microbiology and Infectious Diseases, Erasmus MC, Rotterdam, Netherlands; ^4^ Department of Occupational Health Services, Erasmus MC, Rotterdam, Netherlands; ^5^ Center for Infectious Disease and Vaccine Research, La Jolla Institute for Immunology, La Jolla, CA 92037, USA; ^6^ Department of Medicine, Division of Infectious Diseases and Global Public Health, University of California, San Diego (UCSD), La Jolla, CA 92037, USA.

## Abstract

The severe acute respiratory distress syndrome coronavirus-2 (SARS-CoV-2) Omicron variant (B.1.1.529) is spreading rapidly, even in vaccinated individuals, raising concerns about immune escape. Here, we studied neutralizing antibodies and T-cell responses targeting SARS-CoV-2 D614G (wildtype, WT), and the B.1.351 (Beta), B.1.617.2 (Delta), and B.1.1.529 (Omicron) variants of concern (VOC) in a cohort of 60 health care workers after immunization with ChAdOx-1 S, Ad26.COV2.S, mRNA-1273 or BNT162b2. High binding antibody levels against WT SARS-CoV-2 spike (S) were detected 28 days after vaccination with both mRNA vaccines (mRNA-1273 or BNT162b2), which significantly decreased after 6 months. In contrast, antibody levels were lower after Ad26.COV2.S vaccination but did not wane. Neutralization assays with infectious virus showed consistent cross-neutralization of the Beta and Delta variants, but neutralization of Omicron was significantly lower or absent (up to a 34-fold decrease compared to WT). Notably, BNT162b2 booster vaccination after either two mRNA-1273 immunizations or Ad26.COV.2 priming partially restored neutralization of the Omicron variant, but responses were still up to-17-fold decreased compared to WT. SARS-CoV-2-specific T-cells were detected up to 6 months after all vaccination regimens, with more consistent detection of specific CD4+ than CD8+ T-cells. No significant differences were detected between WT- and variant-specific CD4+ or CD8+ T-cell responses, including Omicron, indicating minimal escape at the T-cell level. This study shows that vaccinated individuals retain T-cell immunity to the SARS-CoV-2 Omicron variant, potentially balancing the lack of neutralizing antibodies in preventing or limiting severe COVID-19. Booster vaccinations are needed to further restore Omicron cross-neutralization by antibodies.

## Teaser

Vaccination-induced SARS-CoV-2-specific CD4+ and CD8+ T-cells targeting the ancestral Spike protein cross-recognize the Omicron variant.

## INTRODUCTION

The severe acute respiratory distress syndrome coronavirus-2 (SARS-CoV-2) Omicron variant (B.1.1.529) is characterized by a high number of mutations in the spike (S) protein that have immune evasive potential. Based on transmission characteristics and immune evasion, the World Health Organization (WHO) designated Omicron as a novel variant of concern (VOC). The Omicron variant has been identified worldwide and models have predicted rapid surges of cases that could surpass earlier peaks (*1*). More data are needed to understand the Omicron disease severity profile, and how severity is impacted by vaccination and pre-existing immunity (*2*).

The large number of mutations and deletions in the Omicron S protein include alterations in the receptor binding domain (RBD), the main target of neutralizing antibodies responsible for host cell entry (*3*–*5*). It was previously shown for the Beta (B.1.351) and Omicron variant that mutations within the RBD (*6*–*8*) and the N-terminal domain (*5*, *9*) can lead to partial escape from neutralizing antibodies. Indeed, there is a concerning reduction in neutralizing antibody titers against Omicron compared to D614G in convalescent and vaccinated individuals, which can be partially restored by booster vaccination (*10*–*14*). Additionally, initial data show that Omicron is resistant to most antibodies authorized for clinical use (*15*, *16*).

Thus far, neutralizing antibodies are regarded the main correlate of protection against severe COVID-19 (*17*, *18*). The relative contribution of virus-specific T-cells is more difficult to decipher. SARS-CoV-2-specific T-cells clear infected cells, thereby contributing to the reduction of viral replication, potentially limiting pathogenicity (*19*). Previous studies on the impact of mutations in the S protein on T-cell recognition show that VOC S proteins are equally recognized by S-specific T-cells induced by mRNA- (*8*, *20*, *21*) and adenovirus-based (*22*) vaccines. Recent studies on T-cell recognition of the Omicron variant show similar results (*23*–*26*). Although the numerous mutations in the S protein in the Omicron variant potentially disrupt multiple T-cell epitopes (*27*), this negative impact is likely to be significantly smaller than the effect on neutralizing antibody epitopes.

Five vaccines have now been authorized for use in Europe by the European Medicines Agency (EMA); mRNA-based (mRNA-1273 [Moderna], BNT162b2 [Pfizer]), adenovirus vector-based (ChAdOx-1 S [Astrazeneca], Ad26.COV2.S [Janssen]) and the protein-based NVX-CoV2373 (Novavax). These vaccines all use the SARS-CoV-2 S protein of the ancestral strain as template for design, induce robust immune responses, and protect from developing severe coronavirus disease-2019 (COVID-19) (*28*–*32*). However, vaccine efficacy differs and is affected by waning of antibodies and the emergence of variants (*18*, *33*–*35*). In general, boosting immune responses by additional vaccinations restores antibody and T-cell responses, but clinical efficacy and protection against Omicron remains to be determined.

Here, we analyzed humoral and cellular immune responses early and late (up to 6 months) after vaccination with ChAdOx-1, S, Ad26.COV2.S, mRNA-1273 or BNT162b2 and performed in-depth analyses of cross-reactivity of neutralizing antibodies and T-cells against against the D614G (wildtype, WT), B.1.351 (Beta), B.1.617.2 (Delta), and B.1.1.529 (Omicron) variants. Additionally, we assessed cross-recognition of variants by neutralizing antibodies and T-cells after booster vaccination. We found cross-neutralization of the Delta and Beta variant in all vaccinated and convalescent individuals, but neutralization of Omicron was consistently lower or even absent. Booster vaccination restored Omicron-specific neutralization, but still at significantly lower levels compared to WT. All VOC, including Omicron, were equally recognized by both CD4+ and CD8+ T-cells.

## RESULTS

### Cohort description

**Fig. 1.**
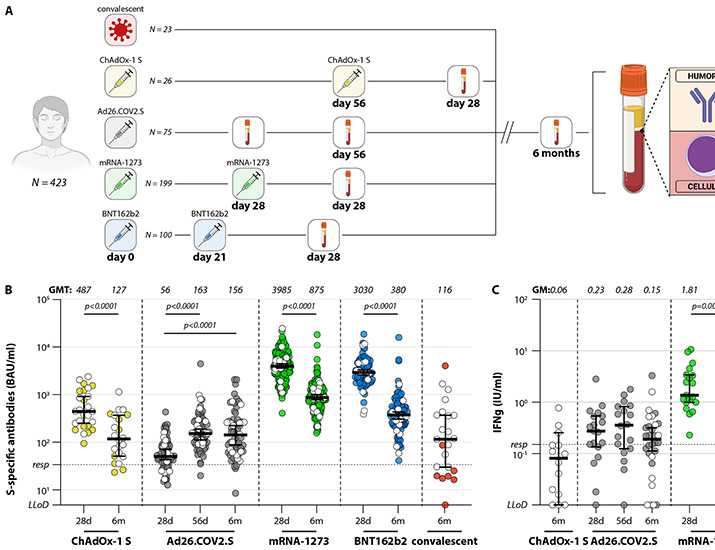
**Study design and detection of virus-specific binding antibodies and T-cells**. (A) Diagram of the number of included participants and study groups. A total of N=423 participants were included for the analysis of binding antibodies and T-cell responses, responses were measured early and late after completion of the vaccination regimen. Binding antibodies were also assessed in N=23 convalescent participants. (B) Levels of binding S-specific antibodies early (28 days after second vaccination, or 56 days after Ad26.COV2.S vaccination) and late (6 months after completion of vaccination regimen) after vaccination or infection. LLoD is 4.81 BAU/ml, responder (resp) cut-off is 33.8 BAU/ml (dotted line). Geometric mean titers are indicated above the graph. (C) IFN-ɣ levels in plasma after stimulation of whole-blood with peptide pools spanning the S protein (Ag2, QIAGEN) early and late after ChAdOX-1 S, Ad26.COV2.S, or mRNA-1273 vaccination, ChAdOx-1 S responses were exclusively measured at 6 months). LLoD is 0.01 IU/ml, responder cut-off is 0.15 IU/ml. Geometric means are indicated above the graph. Comparisons of timepoints within study groups were performed by paired *t* test. White symbols represent samples selected for in-depth analyses. LLoD = lower limit of detection, S = Spike, BAU = binding arbitrary units, GMT = geometric mean titer, d = days, m = months, IFN-ɣ = interferon gamma.

To assess binding antibody and T-cell responses after different vaccination regimens, we collected serum and peripheral blood mononuclear cells (PBMC) from N=400 participants, of which N=26 received two doses ChAdOx-1 S, N=75 received a single dose Ad26.COV2.S, N=199 received 2 doses of mRNA-1273, and N=100 received 2 doses of BNT162b2. Additionally, we measured responses in N=23 plasma donors with a confirmed WT SARS-CoV-2 infection (convalescent). Binding antibodies were assessed early (28 days after second-dose BNT162b2, mRNA-1273 or ChAdOx-1, or 56 days after single dose Ad26.COV2.S) and late (6 months) after vaccination. T-cell responses in whole-blood were assessed at 56 days and 6 months after vaccination (Ad26.COV2.S, N=31), 28 days and 6 months after second vaccination (mRNA-1273, N=39), or exclusively at 6 months after vaccination (ChAdOx-1 S, N=14). Variant-specific virus neutralization and T-cells were performed in a selection of N=15 participants from each vaccination group at the early and late timepoint. The study design is shown in **Fig. 1A**; participant characteristics are summarized in **Table 1**.

**Table 1.** Participant characteristics.

**Cohort**		**N**	**Age (median years, 95% CI)**	**Female (N, %)**	**Vaccination interval** **(median days, 95% CI)**
Convalescent		23	48 (42-58)	4 (17%)	N/A
	*In-depth*	*15*	*53 (42-62)*	*1 (7%)*	*N/A*
ChAdOx-1 S		26	63 (62-64)	17 (61%)	56 (56-70)
	*In-depth*	*15*	*63 (62-64)*	*9 (60%)*	*56 (56-70)*
Ad26.COV2.S		75	37 (32-43)	62 (83%)	N/A
	*In-depth*	*15*	*38 (32-50)*	*13 (87%)*	*N/A*
mRNA-1273		199	41 (39-44)	156 (78%)	28 (28-28)
	*In-depth*	*15*	*34 (27-40)*	*12 (80%)*	*25 (25-28)*
BNT162b2		100	40.5 (37-45)	66 (66%)	21 (21-21)
	*In-depth*	*15*	*46 (36-53)*	*11 (73%)*	*22 (21-25)*

Participants in the ChAdOx-1 S group were significantly older than participants in other groups (p<0.0001, Kruskal-Wallis with multiple comparisons); other groups were comparable. Additionally, the intervals between first and second vaccination were different for all groups that received two vaccines: median interval for (1) ChAdOx-1 S was 56 days, (2) mRNA-1273 was 28 days, and (3) BNT162b2 was 21 days.

### Vaccine-induced antibodies wane after 6 months, except in Ad26.COV2.S vaccinated HCW

Highest levels of S-specific binding antibodies were detected 28 days after full vaccination with mRNA-1273 or BNT162b2 (geometric mean titer [GMT] of 3985 and 3030 BAU/ml, respectively). A significant reduction in GMT was observed after 6 months in both groups (**Fig. 1B**). Both adenovirus vector-based vaccines induced significantly lower binding antibody titers at 28 days (ChAdOx-1 S) or 56 days (Ad26.COV2.S) after vaccination (GMT of 127 and 163 BAU/ml). Whereas a significant drop in antibody levels was observed in ChAdOx-1 S-vaccinated healthcare workers (HCW), the levels remained stable in Ad26.COV2.S-vaccinated HCW. Despite the levels being stable in Ad26.COV2.S-vaccinated HCW, after 6 months the antibody levels were still significantly higher in HCW vaccinated with an mRNA-based vaccine. Interferon (IFN)-ɣ levels were analyzed in ChAdOx-1 S-, Ad26.COV2.S, and mRNA-1273-vaccinated participants after stimulation of whole-blood as a measure for T-cell activity. mRNA-1273-vaccinated HCW had the highest T-cell responses, but significant decreases were observed 6 months after vaccination. T-cell responses remained stable up to 6 months after Ad26.COV2.S vaccination (**Fig. 1C**).

### Significant reduction in Omicron neutralization in convalescent and vaccinated participants

**Fig. 2.**
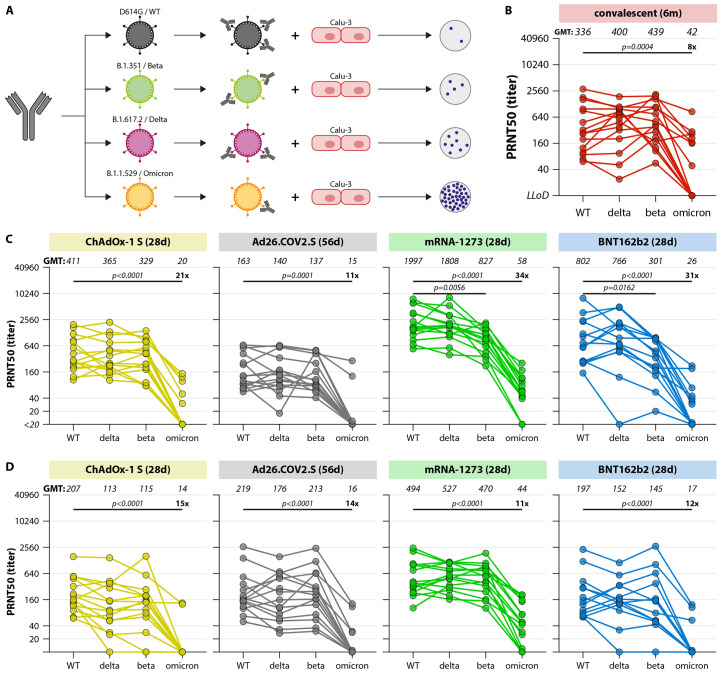
SARS-CoV-2 variant-specific neutralizing antibody responses. (A) Schematic overview of the infectious virus PRNT50 assay. Serum samples were pre-incubated with 400 plaque forming units infectious virus for 1 hour, transferred to Calu-3 cells, and N-positive plaques were counted after 8 hours. A PRNT50 (50% reduction of plaques) was calculated by a proportionate distance formula. (B-D) Levels of neutralizing antibodies in convalescent donors after 6 months (B), early after completion of the vaccination regimen (C), and late after completion of the vaccination regimen (D). N=15 participants were included per vaccination regimen. The lowest serum dilution tested was 1:20, undetectable PRNT50 values (<20) were set at a PRNT50 of 10. Geometric mean titers are indicated above the graphs, fold change reductions are indicated and calculated by dividing the D614G GMT by the Omicron GMT. Comparisons of VOC-specific responses within study groups were performed by Friedman test with multiple comparisons. WT = wildtype, d = days, m = months, PRNT50 = plaque reduction neutralization titer – 50%,

Antibody functionality was measured using an infectious virus neutralization assay with passage 3 SARS-CoV-2 viruses D614G (WT), B.1.351 (Beta), B.1617.2 (Delta) and B.1.1.529 (Omicron) (**Fig. 2A**). Human airway Calu-3 cells were used for virus propagation and neutralization assays because SARS-CoV-2 enters these cells using the TMPRSS2-mediated entry pathway (*36*–*39*). This entry pathway is used in vivo, and prevents adaptations in S, commonly observed in Vero cells. At 28 days post vaccination, mRNA-1273 vaccination elicited highest plaque reduction neutralization titers-50% (PRNT50) against WT and all variants, followed by BNT162b2, ChAdOx-1 S, and Ad26.COV2.S vaccination. Delta and Beta variants were neutralized at slightly lower PRNT50, especially after vaccination with mRNA-based vaccines, and a significant reduction of PRNT50 to Omicron was observed in all groups. Notably, 13/15 participants in the Ad26.COV2.S group did not neutralize the Omicron variant (**Fig. 2C**).

At 6 months post vaccination, neutralizing antibodies against WT waned after ChAdOx-1 S, mRNA-1273 and BNT162b2 vaccination, but remained stable after Ad26.COV2.S vaccination (**Fig. 2C****, ****2D**). Differences in GMT PRNT50 values for the different vaccine groups were smaller at this point. An 11 to 15-fold reduction in PRNT50 to Omicron led to low or completely absent neutralizing antibody levels in all groups. An 8-fold reduction in Omicron neutralization was observed in the selection of 15 convalescent (after WT SARS-CoV-2 infection) sera (**Fig. 2B**). Neutralizing antibodies to the WT, Beta and Delta variants correlated well to measured binding antibody levels, whereas a lower (but significant) correlation was observed between Omicron-specific neutralizing antibodies and binding antibody levels (**Figure S1**).

### Vaccination-induced S-specific T-cells equally recognized VOC including Omicron

**Fig. 3.**
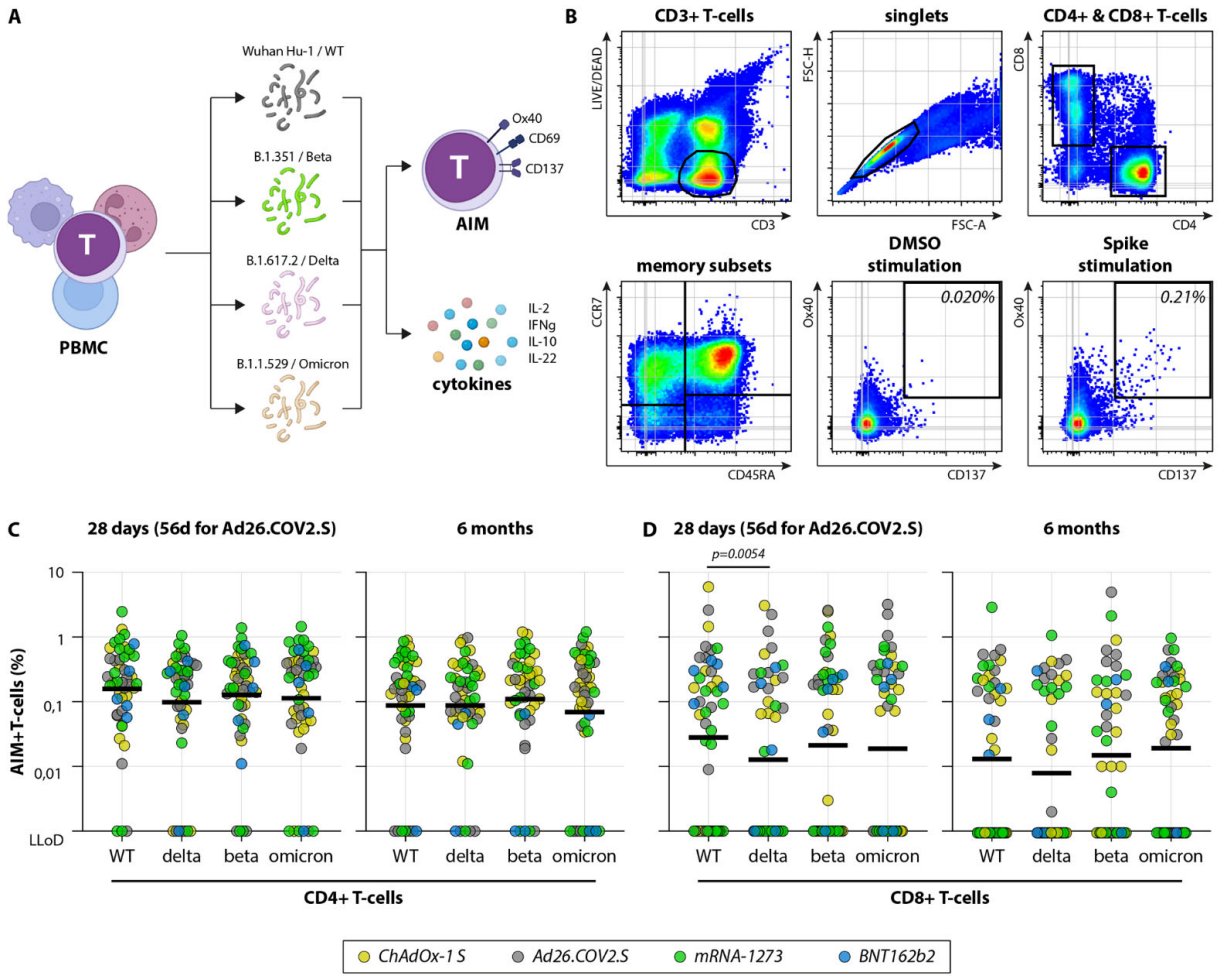
SARS-CoV-2 variant-specific T-cell responses. (A) Schematic overview of AIM assay. PBMC were stimulated with different overlapping peptide pools for 20 hours, followed by measurement of up-regulation of activation markers by flow cytometry and cytokines in cell culture supernatant. (B) SARS-CoV-2-specific T-cells were detected by flow cytometry. (C-D) Up-regulation of AIM early and late after completion of the vaccination regimen on CD4+ (C) and CD8+ (D) T-cells. Percentages indicate the percentage of AIM+ T-cells after subtraction of observed background in a DMSO stimulation, bars indicate the means. N=49 participants were analyzed over various timepoints. Comparisons of VOC-specific responses within study groups were performed by Friedman test with multiple comparisons. LLoD = lower limit of detection, PBMC = peripheral blood mononuclear cells, AIM = activation-induced markers, d = days.

Besides neutralizing antibodies, we assessed the presence of S-specific CD4+ and CD8+ T-cells in the selected participants early and late after vaccination (**Fig. 3**). For the selected BNT162b2-vaccinated individuals a limited set of PBMC was available (N=5 out of 15). PBMC were stimulated with either overlapping peptide pools representing the full-length WT S protein, or peptide pools based on the S proteins of respectively the Beta, Delta or Omicron variants (**Fig. 3A**). After stimulation, the specific production of 13 different cytokines was measured in cell culture supernatants, and AIM expression on CD4+ (OX40 and CD137) and CD8+ (CD69 and CD137) T-cells was measured by flow cytometry (**Fig. 3B**). Based on the production of IL-2, IFN-γ, IL-10 and IL-22 in culture supernatants (upon S-specific peptide stimulation compared to a DMSO control stimulation), S-specific T-cells were detected in the majority of vaccinated individuals, early and late after vaccination (**Figure S3**). No differences in cytokine production were detected after stimulation with either the WT or Omicron S peptide pool.

Based on AIM expression (OX40+CD137+) after WT S peptide pool stimulation of samples obtained at 28 days after any vaccination, we detected S-specific CD4+ T-cells in 94% of the vaccine recipients (46/49). We did not observe significant differences between CD4+ T-cell responses to WT, Delta, Beta or Omicron S peptide pools early or late after vaccination with any vaccine (**Fig. 3C**). When considering the vaccine regimens separately, significant differences between variant-specific T-cell responses were not detected (**Figure S4**).

S-specific CD8+ T-cells were more difficult to detect, likely because we used peptide pools containing overlapping 15-mer peptides (whereas 8- to 10-mers would be optimal for HLA class I binding). Based on AIM expression (CD69+CD137+) after WT S peptide pool stimulation of samples obtained at 28 days after any vaccination, we detected S-specific CD8+ T-cells in 63% of the vaccine recipients (31/49). We did not observe significant differences between CD8+ T-cell responses to WT, Delta, Beta or Omicron S peptide pools early or late after vaccination (**Fig. 3D**). There was one exception, Delta-specific CD8+ T-cells were significantly lower compared to WT-specific CD8+ T-cell responses in samples obtained 28 days after vaccination. When considering the vaccine regimens separately, significant differences between variant-specific T-cell responses were not observed (**Figure S5**).

### Increased cross-reactivity of neutralizing antibodies and T-cells against Omicron after boost

**Fig. 4.**
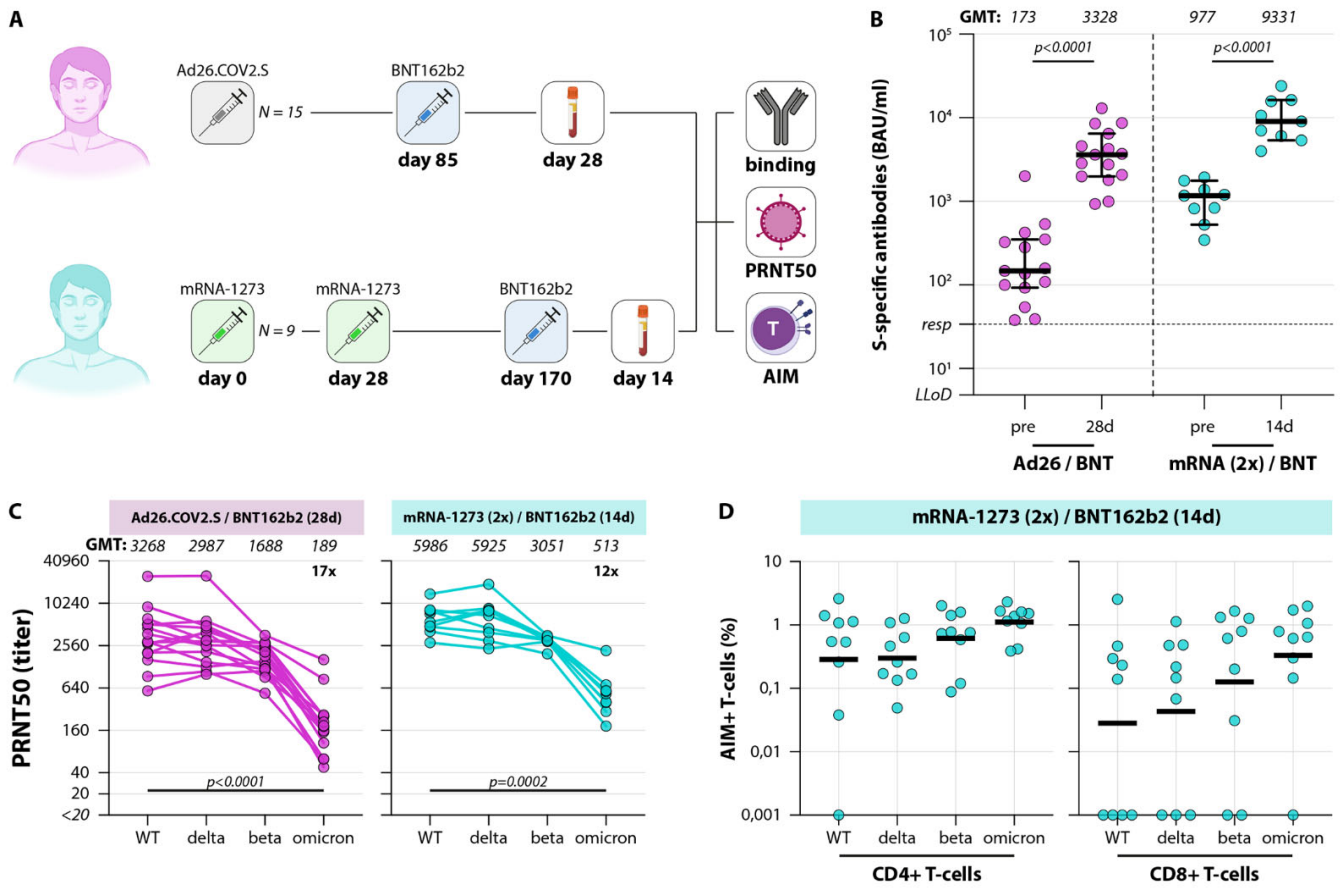
Variant-specific immune responses early after booster vaccination. (A) Diagram of the number of included participants in the booster analysis. A total of N=24 participants were included for the analysis of binding antibodies, variant-specific neutralizing antibodies, and variant-specific T-cell responses. N=15 participants were primed with one shot Ad26.COV2.S, N=9 participants were primed with two shots mRNA-1273, all participants were analyzed throughout the panels. Responses were measured early after BNT162b2 booster vaccination. (B) Levels of binding S-specific antibodies early after booster vaccination. LLoD is 4.81 BAU/ml, responder (resp) cut-off is 33.8 BAU/ml (dotted line). Geometric mean titers are indicated above the graph. Comparisons of timepoints within study groups were performed by paired *t* test. (C) Levels of neutralizing antibodies in boosted donors. The lowest serum dilution tested was 1:20, undetectable PRNT50 values (<20) were set at a PRNT50 of 10. Geometric mean titers are indicated above the graphs, fold change reductions are indicated and calculated by dividing the D614G GMT by the Omicron GMT. (D) Up-regulation of AIM on CD4+ T-cells and CD8+ T-cells in boosted donors. Percentages indicate the percentage of AIM+ cells after subtraction of observed background in a DMSO stimulation, bars indicate the means. Comparisons of VOC-specific responses within study groups were performed by Friedman test with multiple comparisons. BAU = binding arbitrary units, GMT = geometric mean titer, PRNT50 = plaque reduction neutralization titer – 50%, LLoD = lower limit of detection, AIM = activation-induced markers, d = days.

Finally, we performed an analysis of neutralizing antibody and T-cell responses to the different SARS-CoV-2 variants after booster vaccination. HCW vaccinated with either one dose of Ad26.COV2.S (N=15) (*40*) or 2 doses mRNA-1273 (N=9) were included; both groups were boosted with BNT162b2, either at 85 days after primary vaccination (Ad26.COV2.S) or at 214 days after completion of the primary vaccination series (mRNA-1273) (**Fig. 4A**). Specimen were collected 28 days (Ad26.COV2.S/BNT162b2) or 14 days (2x mRNA-1273/BNT162b) after boost. Participant characteristics are described in **Table 2**.

**Table 2.** Booster participant characteristics.

**Cohort**	**N**	**Age (median years, 95% CI)**	**Female (N, %)**	**Vaccination interval** **(median days, 95% CI)**
Ad26.COV2.S / BNT162b2	15	37 (28-44)	10 (67%)	88 (79-88)
2x mRNA-1273 / BNT162b2	9	29 (27-40)	7 (78%)	214 (214-219)

Boosting with BNT162b led to rapid recall responses in both Ad26.COV2.S and mRNA-1273 primed individuals, and a significant increase in binding antibody levels was observed (**Fig. 4B**). Binding antibody levels in the mRNA-1273 boosting regimen reached the highest levels (GMT of 9331 versus 3328 in the Ad26.COV2.S-primed individuals). PRNT50 after boost were also higher in the mRNA-1273 primed individuals compared to the Ad26.COV2.S-primed individuals (**Fig. 4C**). When comparing neutralization of the different VOC, no drop in neutralization of Delta, a two-fold drop in neutralization of Beta, but a significant 17- and 12-fold drop in neutralization of Omicron was still observed. However, neutralization of Omicron was observed or restored in all boosted individuals (**Fig. 4C**). Similar to the observations upon primary vaccination regimens, no reduced T-cell responses to variants (including Omicron) were detected after boost (**Fig. 4D**).

## DISCUSSION

Here, we demonstrate the resistance of the SARS-CoV-2 Omicron (B.1.1.529) variant to neutralizing antibodies induced by mRNA-based or adenovirus vector-based vaccination. We observed up to 30-fold reductions of Omicron neutralizing titers when compared to WT SARS-CoV-2. Lower neutralizing antibody levels were previously associated with an increased risk of SARS-CoV-2 infection (*18*), and a higher burden of disease. In contrast, we show that SARS-CoV-2-specific T-cell responses were minimally affected by mutations in the Omicron S protein. T-cell activity is thought to confer protection from severe disease, but it remains to be determined whether this is sufficient in the absence of a potent neutralizing antibody response. Importantly, a single BNT162b2 booster immunization induced a substantial increase in Omicron-specific neutralization after priming with either Ad26.COV2.S or mRNA-1273 in the short term. Neutralization of Omicron was partially restored in individuals without cross-reactive neutralizing antibodies prior to boosting, and cross-recognition of the different variants by CD4+ and CD8+ T-cells was maintained after booster vaccination.

We observed high, but transient, levels of SARS-CoV-2-specific binding antibodies after mRNA-based vaccination. Despite the waning of binding antibody levels in vaccinees, affinity maturation of antibodies and a continued increase in SARS-CoV-2-specific memory B cells was previously described between 3 and 6 months after mRNA vaccination (*41*). These memory B cells are generated in prolonged germinal center reactions (*42*), and are capable of mounting a rapid recall response upon re-encountering the S protein, either by natural infection or booster vaccination. In contrast, we observed that Ad26.COV2.S vaccination led to antibody responses which showed minimal waning at 6 months, in line with recent findings by Barouch *et al*. (*43*). This suggests that upon Ad26.COV2.S vaccination maturation of B cells occurs without further short-term boosting. Neutralizing antibodies are regarded the main correlate of protection against infection with SARS CoV-2 (*17*, *18*). The capacity of sera from vaccinated individuals to cross-neutralize the Omicron variant in our study was reduced up to 30-fold (*9*, *10*, *13*–*16*, *44*, *45*). To be protected against symptomatic COVID-19 caused by Omicron, affinity maturation will be essential for the acquisition of broader neutralizing activity of RBD-binding antibodies that were previously formed (*46*, *47*). Booster vaccination of Ad26.COV2.S- and mRNA-1273-primed individuals quickly increased S-specific antibodies and restored Omicron neutralization (*11*, *12*, *48*), however these levels were still significantly lower than neutralization of WT. Whether booster vaccinations increase the breadth of the response remains to be determined, as well as whether antibody responses targeting variants are maintained long-term (*43*).

Multiple assays to determine the neutralizing capacity of sera are currently employed (*49*), and the variety of protocols hinders a direct head-to-head comparison of different studies. We studied VOC-specific neutralization using infectious virus plaque reduction assays. It is known that SARS-CoV-2 propagation on VeroE6 cells, a cell-line routinely used for SARS-CoV-2 neutralization assays, can lead to mutations or deletions in the multibasic cleavage site in the S protein (*36*–*39*). We therefore grew the viral stocks and performed the neutralization assays on the TMPRSS2 expressing human airway cell-line Calu-3.

It was previously shown that vaccine-induced T-cells targeting the ancestral S protein demonstrate minimal reductions in frequency and magnitude against the Alpha, Beta, Gamma and Delta variants (*8*, *21*, *22*, *25*, *41*, *50*). Recently, preservation of T-cell reactivity with the Omicron S protein was also demonstrated (*23*–*26*). Here, we performed a comprehensive analysis of SARS-CoV-2-specific T-cell responses in vaccinated individuals early and late after receiving ChAdOx-1 S, Ad26.COV2.S, mRNA-1273, or BNT162b2 vaccination. In contrast to the humoral response, we showed durable cellular immune responses that persisted for at least 6 months after either mRNA-based or adenovirus vector-based vaccination. Vaccine-induced S-specific CD4+ T-cells were detected in 94% of the vaccine recipients, whereas we could detect S-specific CD8+ T-cell responses in 63% of the vaccine recipients. In line with previous studies into VOC-specific T-cell responses, vaccine-induced T-cells equally recognized the WT, Beta, Delta, and Omicron variant independent of timing or vaccination regimen, although we only included 5 BNT162b2-vaccinated participants so these results were interpreted with caution. After peptide pool stimulation, we detected production of typical Th1 (IFN-γ and IL-2), Th2 (IL-10) and Th17 (IL-22) cytokines, without differences between WT and Omicron S stimulation. This fits well with the observation that although Omicron has numerous mutations in the S protein, T-cell epitopes are minimally affected. Variant-specific CD4+ and CD8+ T-cell responses were maintained after booster vaccination.

Our study has several limitations: first, the sample size for which in-depth T cell profiling was performed was limited to 15 donors per vaccination regimen, and we measured lower levels of CD8+ T-cells likely due to the use of 15-mer peptides. Additional studies with smaller peptides (8- to 10-nucleotide oligomers) predicted or shown to bind human leukocyte antigen class I are advised to specifically study VOC cross-reactive CD8+ T cell responses. Furthermore, the data post booster vaccination is limited, and longevity of variant-specific immune responses post booster vaccination remain to be determined. Finally, this study is skewed toward healthy young-adult participants (with the exception of ChAdOx-1 S-vaccinated individuals).

Although in-depth analyses were performed in a relatively small number of subjects, we show that vaccinated individuals retain T-cell immunity to the Omicron variant, potentially balancing the lack of neutralization antibodies in preventing or limiting severe COVID-19. Furthermore, the fact that booster vaccinations restored and increased the neutralizing capacity against Omicron supports the call for immediate booster campaigns. In the near future, variant-specific booster vaccines may be required to optimally skew the immune responses toward emerging viruses.

## MATERIALS AND METHODS

### Study design

A prospective cohort study of health care workers (HCW) at Erasmus MC was initiated in 2020, with a focus on immunity against SARS CoV-2 after symptomatic presentation to the occupational health services. From January 2021, HCW in the Netherlands were offered vaccination with one of the 4 approved vaccines in the Netherlands (ChAdOx-1 S, Ad26.COV2.S, mRNA-1273 and BNT162b2), participants of the HCW study were invited for the follow up study in which immune responses upon vaccination were studied. From December 2021, HCW in the study were invited for a follow up study after booster vaccination with BNT162b2. A single-blind, multicenter, randomized controlled trial (SWITCH trial) was performed to study immune responses after heterologous booster vaccination following a priming vaccination with Ad26.COV2.S (*40*). Finally, we performed a study in which PCR-confirmed COVID-19 patients were recruited to participate as plasma donors (ConCOVID) (*51*). A combination of samples was used for a complete immunological assessment of SARS-CoV-2-specific immune responses after vaccination targeting different variants of concern.

### Ethics statement

Samples from three different trials (HCW, ConCOVID, and SWITCH) were analyzed in the scope of this study (**Table S1**). The HCW study was approved by the institutional review board of the Erasmus MC (medical ethical committee, MEC-2020-0264) (*8*). The ConCOVID trial was also approved by the institutional review board of the Erasmus MC (MEC 2020-0228). The study was registered at clinicaltrials.gov (NCT04342182) (*51*). The SWITCH trial was approved by the institutional review board of the Erasmus MC (MEC 2021-0132) and local review boards of participating centres. The study was registered at clinicaltrials.gov (NCT04927936) (*40*). All studies adhere to the principles of the Declaration of Helsinki, and written informed consent was obtained from every participant, patient or legal representative.

### Study design and sample collection

Serum and PBMC samples from all vaccinated participants of the HCW study were obtained 28 days and 6 months after second vaccination (with the exception of Ad26.COV2.S vaccinated HCW, samples were obtained 56 days after first vaccination). HCW were vaccinated with (1) ChAdOx-1 S / ChAdOx-1S (56 day interval), (2) Ad26.COV2.S, (3) mRNA-1273 / mRNA-1273 (28 day interval), or BNT162b2 / BNT162b2 (21 days interval) (**Fig. 1A**). Additional serum and PBMC samples were obtained from 9 mRNA1273 / mRNA-1273-primed participants at 14 days after booster vaccination with BNT162b2. Participants of the SWITCH trial were vaccinated with Ad26.COV2.S, followed by a BNT162b2 boost (85 day interval) (*40*). Serum samples were obtained 28 days after the booster vaccination (**Fig. 4A**). Participants of the HCW and SWITCH trial had no history of previous SARS-CoV-2 infection, as confirmed by absence of N-specific antibodies and/or S-specific antibodies pre-vaccination at baseline. Serum samples from convalescent PCR-confirmed COVID-19 patients were obtained 6 months after infection in the scope of the ConCOVID study, in which study participants were recruited to participate as plasma donors (*51*). These patients were infected during the first wave in 2020, all with a D614G SARS-CoV-2. In-depth neutralization and T-cell assays were performed on a selection of N=15 participants from each group (**Table 1**, **Table 2**). The selection of participants for in-depth analyses was based on availability of longitudinal PBMC samples and the distribution of antibody responses (**Fig. 1B****, ****1C**).

### Serum and PBMC isolation

Serum was collected in 10-ml tubes without anticoagulant, centrifuged at 2500 rpm for 15 min, aliquoted, and stored at −20°C for further experiments. PBMCs were isolated from blood collected in K_3_EDTA or Lithium Heparin tubes by density gradient centrifugation. Briefly, blood was layered on a density gradient (Lymphoprep, STEMCELL Technologies), and PBMCs were separated by centrifuging at 2000 rpm for 30 min. PBMCs were washed four times in PBS and subsequently frozen in liquid nitrogen in 90% fetal bovine serum (FBS) with 10% dimethyl sulfoxide (DMSO; Honeywell) until use in stimulation assays.

### Detection of S-specific binding antibodies

Binding antibodies against the SARS-CoV-2 Spike (S) protein were measured by Liaison SARS-CoV-2 TrimericS IgG assay (DiaSorin, Italy), with a lower limit of detection of 4.81 BAU/ml and a cut-off for positivity at 33.8 BAU/ml. The assay was performed following the manufacturer’s instructions.

### IFN-ɣ release assay (IGRA)

The SARS-CoV-2-specific T cell response was measured by commercially available IFN-ɣ Release Assay (IGRA, QuantiFERON, Qiagen) in whole blood as previously described and following the manufacturer’s description (*52*). In short, heparinized whole blood was incubated with three different SARS-CoV-2 antigens for 20-24h using a combination of peptides stimulating both CD4^+^ and CD8^+^ T-cells (Ag1, Ag2, Ag3, QuantiFERON, QIAGEN). After incubation, plasma was obtained and IFN-ɣ production in response to the antigens was measured by ELISA. Results are expressed in IU IFN-ɣ /ml after subtraction of the NIL control values as interpolated from a standard calibration curve. Lower limit of detection in this assay is set at 0.01 IU/ml, responder cut-off is 0.15 IU/m). IFN-ɣ production after stimulation with Ag2, containing peptides covering the S protein, was shown in this study.

### Virus culture and deep-sequencing

SARS-CoV-2 isolates were grown to passage 3 on Calu-3 (ATCC HTB-55) cells in Advanced DMEM/F12 (Gibco), supplemented with HEPES, Glutamax, penicillin (100 IU/mL) and streptomycin (100 IU/mL) at 37°C in a humidified CO_2_ incubator. Infections were performed at a multiplicity of infection (MOI) of 0.01 and virus was harvested after 72 hours. The culture supernatant was cleared by centrifugation at 1000 × g for 5 min and stored at −80°C in aliquots. Stock titers were determined by incubating 10-fold dilutions of virus stock in OptiMEM at 37°C for 1 hour, after which they were transferred onto Calu-3 cells and incubated for 8 hours at 37°C in a humidified CO_2_ incubator. Next, cells were fixed with 4% formalin, permeabilized in 70% ethanol, after which infected cells were stained with polyclonal rabbit anti–SARS-CoV-2 nucleocapsid antibody (Sino Biological) and a secondary goat anti-rabbit IgG AF488 (Invitrogen). Stained plates were scanned on the Amersham Typhoon Biomolecular Imager (channel Cy2; resolution 10 μm; GE Healthcare) and the number of infected cells was determined to calculate the stock titer per milliliter. All work with infectious SARS-CoV-2 was performed in a Class II Biosafety Cabinet under BSL-3 conditions at Erasmus Medical Center.

Viral genome sequences were determined using Illumina deep-sequencing. RNA was extracted using AMPure XP beads and cDNA was generated using ProtoscriptII reverse transcriptase enzyme (New England, BiotechnologyBioLabs) according to the manufacturer’s protocol (*53*). Samples were amplified using the QIAseq SARS-CoV-2 Primer Panel (Qiagen). Amplicons were purified with 0.8x AMPure XP beads and 100ng of DNA was converted to paired-end Illumina sequencing libraries using the KAPA HyperPlus library preparation kit (Roche) with the KAPA unique dual-indexed adapters (Roche) as per manufacturers recommendations. The barcode-labeled samples were pooled and analyzed on an Illumina sequencer V3 MiSeq flowcell (2x300 cycles). Sequences were analyzed using CLC Genomics Workbench 21.0.3. The 614G virus (clade B; isolate Bavpat-1; European Virus Archive Global #026 V-03883) passage 3 sequence was identical to the passage 1 (kindly provided by Dr. Christian Drosten) and no minor variants >20% were detected. The beta variant (clade B.1.351) passage 3 sequence contained two mutations compared the original respiratory specimen: one synonymous mutations C13860T (Wuhan-1 position) in ORF1ab and a L71P change in the E gene (T26456C, Wuhan-1 position). No other minor variants >20% were detected. The delta (clade B.1.617.2) and omicron (clade B.1.1.529) variant passage 3 sequences were identical to the original respiratory specimens and no minor variants >20% were detected. Due to primer mismatches in the S1 region of the omicron spike gene, amplicons 72, 73, 75 and 76 were sequenced at low coverage. Therefore, the S1 regions of the original respiratory specimen and passage 3 virus were confirmed to be identical by Sanger sequencing. The Beta, Delta and Omicron sequences are available on GenBank under accession numbers OM286905, OM287123, and OM287553, respectively.

The beta variant contained the following spike changes: L18F, D80A, D215G, del241-243, K417N, E484K, N501Y, D614G, and A701V. The delta variant contained the following spike changes: T19R, G142D, del156-157, R158G, A222V, L452R, T478K, D614G, P681R and D950N. The omicron variant contained the following spike mutations: A67VS, del69-70, T95I, G142-, del143-144, Y145D, del211, L212I, ins215EPE, G339D, S371L, S373P, S375F, K417N, N440K, G446S, S477N, T478K, E484A, Q493R, G496S, Q498R, N501Y, Y505H, T547K, D614G, H655Y, N679K, P681H, N764K, D796Y, N856K, Q954H, N969K, L981F.

### Detection of neutralizing antibodies by plaque reduction assay

Plaque reduction neutralization tests (PRNT) were performed as described previously (*8*, *54*). Briefly, heat-inactivated sera were two-fold diluted in OptiMEM medium starting at a dilution of 1:10 (or 1:80 for sera known to have more than 2500 BAU/ml S-specific binding antibodies) in 60ul. 400 plaque forming units of different SARS-CoV-2 variants were added to each well in 60ul of virus suspension incubated at 37°C for 1 hour. After 1 hour of incubation, the virus-antibody mixtures were transferred onto the human airway cell line Calu-3 and incubated for 8 hours. After incubation, cells were fixed and plaques were stained with polyclonal rabbit anti–SARS-CoV-2 nucleocapsid antibody (Sino Biological) and a secondary peroxidase-labeled goat anti-rabbit IgG (Dako). Signal was developed by using a precipitate-forming 3,3′,5,5′-tetramethylbenzidine substrate (TrueBlue; Kirkegaard & Perry Laboratories) and the number of infected cells was counted per well by using an ImmunoSpot Image Analyzer (CTL Europe GmbH). The dilution that would yield 50% reduction of plaques (PRNT50) compared with the infection control was estimated by determining the proportionate distance between two dilutions from which an endpoint titer was calculated. Raw data for neutralizing antibodies early after vaccination (**Figure S2A, S2B**), late after vaccination (**Figure S2C, S2D**), 6 months after positive PCR (**Figure S2E, S2F**), or early after booster vaccination (**Figure S2G, S2H**) are included in the supplemental figures. Infection controls and positive serum controls were included on each plate, the NIBSC standard was included in three separate experiments. When no neutralization was observed, we set the PRNT50 one dilution step below the dilution series, i.e., a PRNT50 value of 10.

### Overlapping SARS-CoV-2 Spike peptide pools

SARS-CoV-2 peptides were synthesized as crude material (TC Peptide Lab, San Diego, CA). Overlapping 15-mer by 10 amino acids covering the full-length S proteins from the WT, Beta (B.1.351), Delta (B.1.617.2) and Omicron (B.1.1.529) variants were synthesized and individually resuspended in dimethyl sulfoxide (DMSO) at a concentration of 10–20 mg/mL. Megapools (MP) for each spike variant were generated by pooling aliquots of these individual peptides, undergoing another lyophilization, and resuspending in DMSO at 1 mg/mL (*21*).

### Stimulations for detection of SARS-CoV-2-specific CD4+ and CD8+ T-cells

PBMCs were thawed in Gibco Roswell Park Memorial Institute 1640 medium (Gibco) supplemented with 10% human serum (Sanquin, Rotterdam), penicillin (100 IU/ml; Lonza, Belgium), streptomycin (100 μg/ml; Lonza, Belgium), and 2 mM L-glutamine (Lonza, Belgium; R10H medium) and treated with Benzonase (50 IU/ml; Merck) at 37°C for 30 min. Subsequently, 1 × 10^6^ PBMCs were stimulated with SARS-CoV-2 variant peptide pools at 1 μg/ml per peptide in 200 μl in a 96-well U-bottom plate at 37°C for 20 hours. Cells were additionally stimulated with an equimolar concentration of DMSO (negative control) or a combination of phorbol 12-myristate 13-acetate (50 μg/ml) and ionomycin (500 μg/ml) (positive control). Additionally, PBMC were stimulated with a peptide pool consisting of 176 known peptides for a broad range of HLA subtypes and different infectious agents (including human herpesviruses and influenza virus) as peptide positive control (PepMix CEFX Ultra SuperStim Pool, JPT). After stimulation, cells were stained for phenotypic lymphocyte markers.

### Multiplex detection of cytokines

Cytokines in cell culture supernatants from ex vivo stimulations were quantified using a human T_H_ cytokine panel (13-plex) kit (LEGENDplex, BioLegend) as described previously (*55*). Briefly, cell culture supernatants were mixed with beads coated with capture antibodies specific for IL-5, IL-13, IL-2, IL-6, IL-9, IL-10, IFN-γ, TNF-α, IL-17A, IL-17F, IL-4, IL-21, and IL-22. After staining, cytokines were detected by flow cytometry on a FACSLyric (BD Biosciences). IL-2, IFN-γ, IL-10, and IL-22 were found specifically produced after peptide pool stimulation, other cytokines are not shown.

### Detection of AIM by flow cytometry

Detection of AIM was performed as described previously (*8*). Briefly, cells were stained with the following antibodies in their respective dilutions: anti–CD3-PerCP (1:25, clone SK7, BD), anti–CD4-V50 (1:50, clone L200; BD), anti–CD8–fluorescein isothiocyanate (1:25, clone DK25; Dako), anti–CD45RA-phycoerythrin (PE)–Cy7 (1:50, clone L48; BD), anti–CCR7-BV711, anti–CD69-allophycocyanin (APC)-H7 (1:50, clone FN50; BD), anti–CD137-PE (1:50, clone 4B4-1; Miltenyi), and anti–OX40-BV605 (1:25, clone L106; BD). LIVE/DEAD Fixable Aqua Dead Cell staining was included (1:100, AmCyan; Invitrogen) and acquired on a FACSLyric (BD Biosciences). After setting a time gate, LIVE CD3+ T-cells were gated, singlets were selected, and T-cells were subtyped into CD3+CD4+ and CD3+CD8+ cells. Within the CD4+ and CD8+ T-cells, T_naive_ were defined as CD45RA+CCR7+, T_CM_ as CD45RA-CCR7+, T_EM_ as CD45RA-CCR7-, and T_EMRA_ as CD45RA+CCR7-. S-specific T-cells were detected by co-expression of AIM on CD4+ (OX40 and CD137) or CD8+ (CD69 and CD137) T-cells in the combination of memory subsets (**Fig. 3A**). The DMSO-stimulated sample was used to set the cutoff gate for activation markers. On average, 500,000 cells were acquired per sample.

### Statistical analysis

A comparison of the baseline characteristics age and interval between vaccination between groups was performed by Kruskal-Wallis with multiple comparisons. Reduction in binding and neutralizing antibody levels over time within groups was analyzed by paired *t* test. Different groups were compared by unpaired *t* test. Differences in PRNT50 and T-cell responses to VOC were estimated by Friedman test with multiple comparisons, data was not normally distributed. Multiple comparisons were always performed as the variants versus WT, variants were not compared between themselves. Spearman R was calculated for the correlation between binding and neutralizing antibodies. All statistical analyses were performed on log-transformed data.

### Software

Statistical analyses were performed with Graphpad PRISM version 9.1.2. Expression of AIM was analyzed with FlowJo software version 10.8.1.

## Supplementary Materials

### Other Supplementary Material for this manuscript includes the following:

MDAR Reproducibility Checklist
