# Supplementary material for: Divergent SARS CoV-2 Omicron-reactive T- and B cell responses in COVID-19 vaccine recipients
Source: Sci Immunol. 2022 Feb 3:eabo2202. doi: 10.1126/sciimmunol.abo2202 (PMC8939771; doi:10.1126/sciimmunol.abo2202)
Supplement: Supplementary file 1 — Figs. S1 to S5 Table S1 [file sciimmunol.abo2202_sm.pdf]

Science Immunology
AAAS

Supplementary Materials for

Divergent SARS CoV-2 Omicron-reactive T- and B cell responses in COVID-19 vaccine recipients

Corine H. GeurtsvanKessel *et al.*

Corresponding authors: Corine H. GeurtsvanKessel, c.geurtsvankessel@erasmusmc.nl;
Bart L. Haagmans, b.haagmans@erasmusmc.nl; Rory D. de Vries, r.d.devries@erasmusmc.nl

DOI: 10.1126/sciimmunol.abo2202

The PDF file includes:

Figs. S1 to S5
Table S1

Other Supplementary Material for this manuscript includes the following:

Data file S1

Supplemental material belonging to

Divergent SARS CoV-2 Omicron-reactive T- and B-cell responses in COVID-19 vaccine recipients

Short title: *Omicron-reactive immune responses post vaccination*

Corine H. GeurtsvanKessel[1]*, Daryl Geers[1†], Katharina S. Schmitz[1†], Anna Z. Mykytyn[1†], Mart M Lamers[1†], Susanne Bogers[1], Sandra Scherbeijn[1], Lennert Gommers[1], Roos S.G. Sablerolles[2], Nella N. Nieuwkoop[1], Laurine C. Rijsbergen[1], Laura L.A. van Dijk[1], Janet de Wilde[1], Kimberley Alblas[1], Tim I. Breugem[1], Bart J.A. Rijnders[3], Herbert de Jager[4], Daniela Weiskopf[5], P. Hugo M. van der Kuy[2], Alessandro Sette[5,6], Marion P.G. Koopmans[1], Alba Grifoni[5$], Bart L. Haagmans[1$]*, Rory D. de Vries[1$]*

Affiliations

[1] Department of Viroscience, Erasmus MC, Rotterdam, the Netherlands

[2] Department of Hospital Pharmacy, Erasmus MC, Rotterdam, Netherlands

[3] Department of Medical Microbiology and Infectious Diseases, Erasmus MC, Rotterdam, The Netherlands

[4] Department of Occupational Health Services, Erasmus MC, Rotterdam, Netherlands

[5] Center for Infectious Disease and Vaccine Research, La Jolla Institute for Immunology, La Jolla, CA 92037, USA

[6] Department of Medicine, Division of Infectious Diseases and Global Public Health, University of California, San Diego (UCSD), La Jolla, CA 92037, USA.

[†] Authors contributed equally

[$] Authors contributed equally

Corresponding authors: Corine H. GeurtsvanKessel (c.geurtsvankessel@erasmusmc.nl), Bart L. Haagmans (b.haagmans@erasmusmc.nl) and Rory D. de Vries (r.d.devries@erasmusmc.nl).

Supplemental Figures

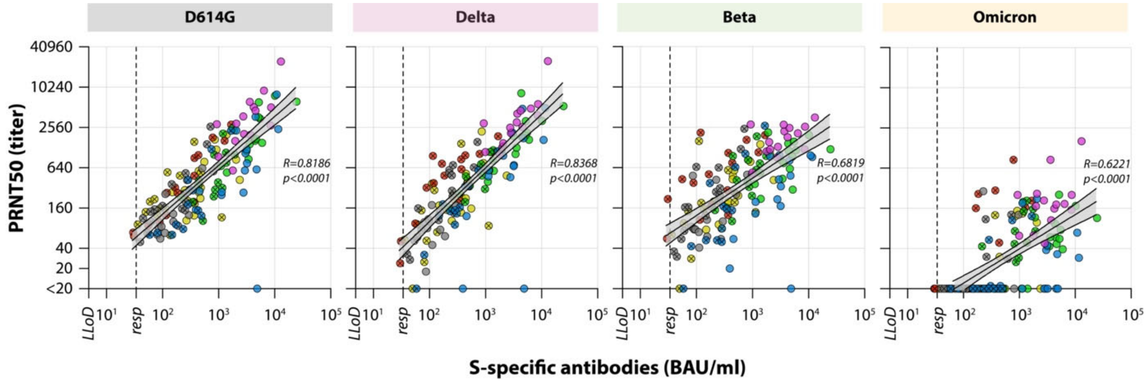

Figure S1. Correlation between neutralizing antibodies and S-specific binding antibodies. Correlation between S-specific biding antibodies early (open symbols) and late (symbols with cross) after vaccination or infection with D614G-specific, Delta-specific, Beta-specific, or Omicron-specific neutralizing antibodies. For S-specific binding antibodies the LLoD is 4.81 BAU/ml, responder (resp) cut-off is 33.8 BAU/ml (dotted line). For neutralizing antibodies, the lowest serum dilution tested was 1:20, undetectable PRNT50 values (<20) were set at a PRNT50 of 10. LLoD = lower limit of detection, BAU = binding arbitrary units, PRNT50 = plaque reduction neutralization titer – 50%. Spearman R was calculated on basis of log-transformed data.

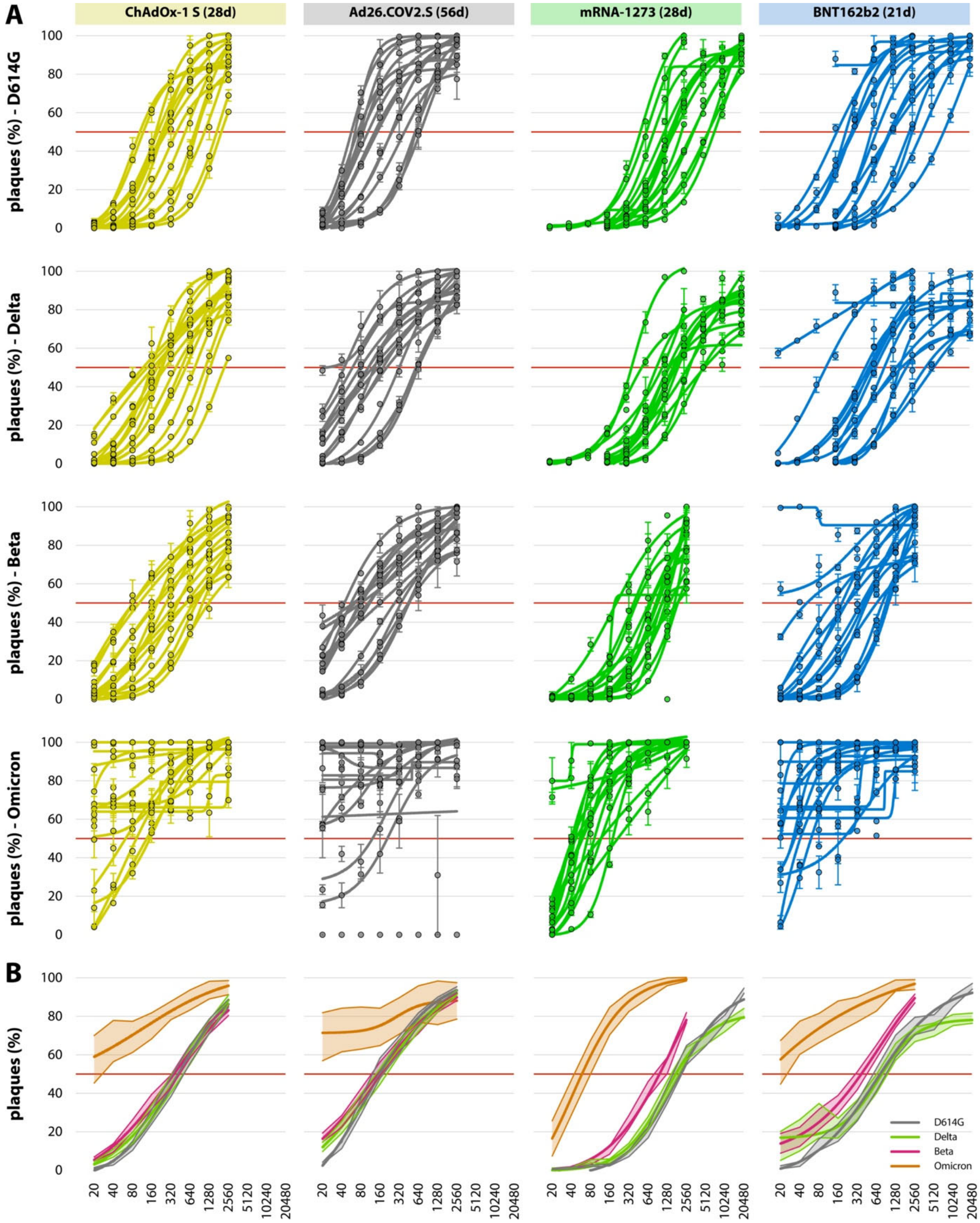

A
ChAdOx-1 S (28d)
Ad26.COV2.S (56d)
mRNA-1273 (28d)
BNT162b2 (21d)
plaques (%) - D614G
plaques (%) - Delta
plaques (%) - Beta
plaques (%) - Omicron
B
plaques (%)
D614G
Delta
Beta
Omicron
20 40 80 160 320 640 1280 2560 5120 10240 20480

40

41

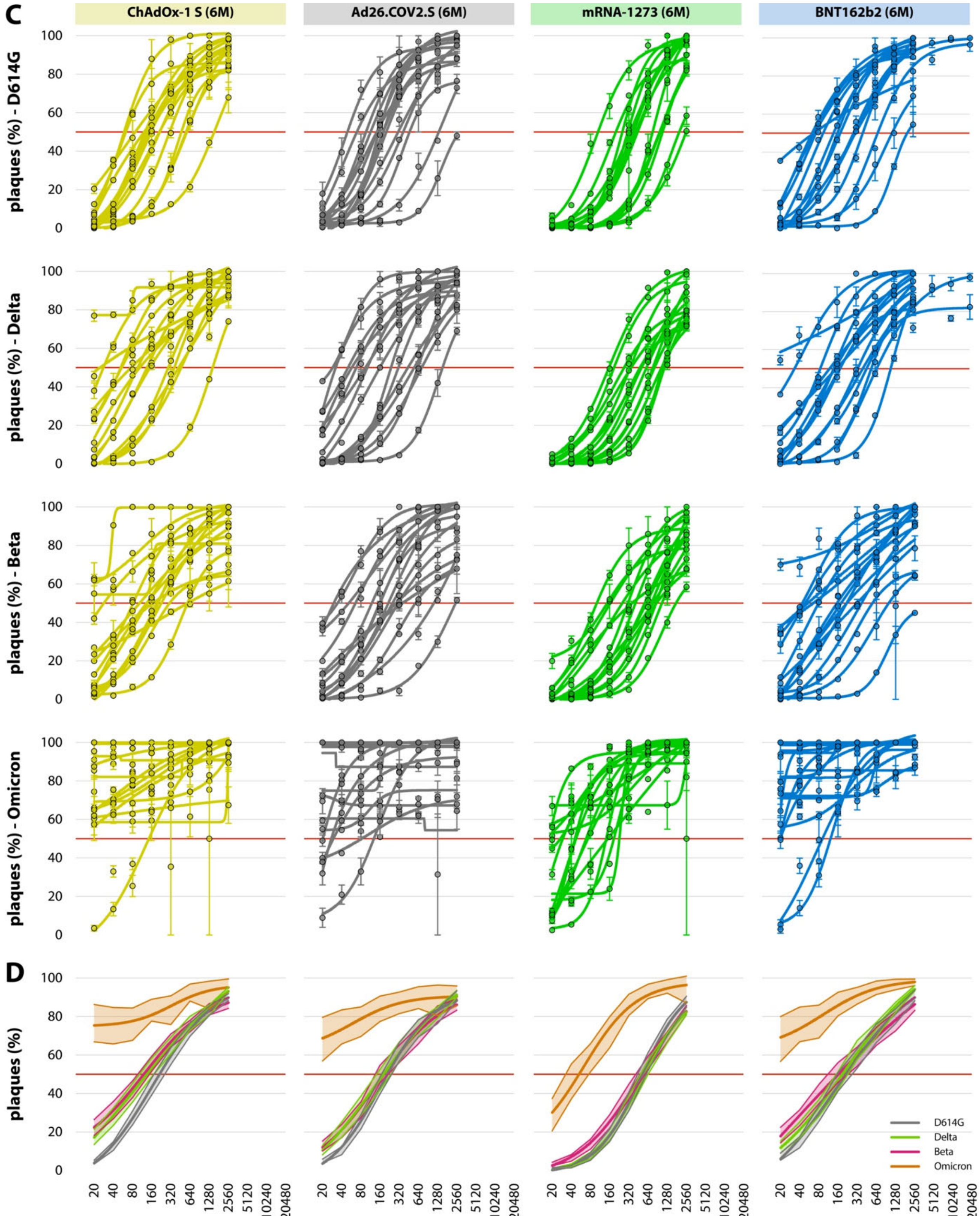
C
ChAdOx-1 S (6M)
Ad26.COV2.S (6M)
mRNA-1273 (6M)
BNT162b2 (6M)
plaques (%) - D614G
plaques (%) - Delta
plaques (%) - Beta
plaques (%) - Omicron
D
plaques (%)
D614G
Delta
Beta
Omicron
0
20
40
60
80
100
20
40
80
160
320
640
1280
2560
5120
10240
20480

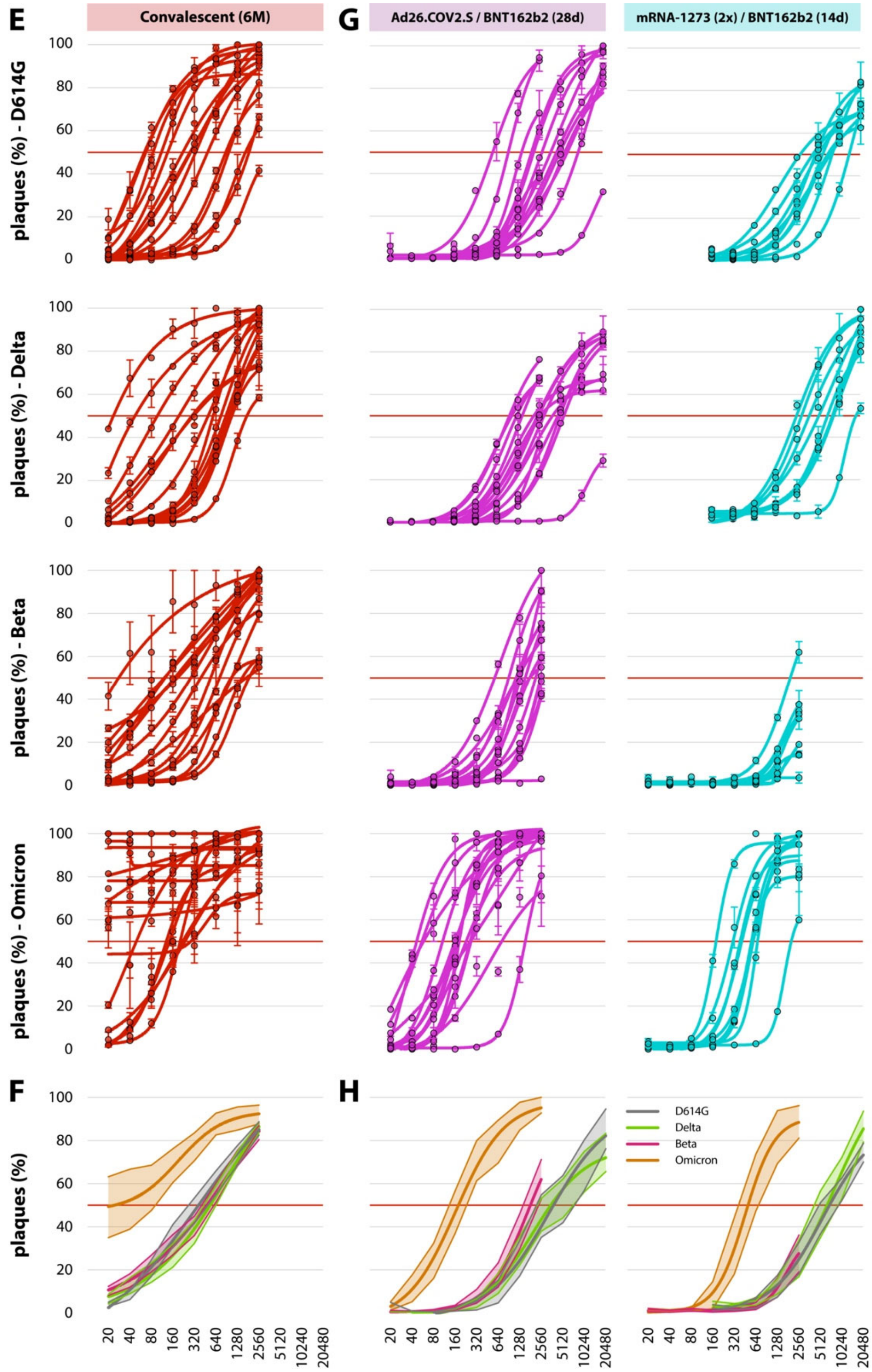

Figure S2. PRNT50 calculations. Graphs in panels A, C, E, and G show per participant log(inhibitor) versus responses curves with four parameter variable slopes based on plaque counts compared to the virus control, for the four different variants. Panels B, D, F, and H show the average curves from all participants with the 95% CI. Neutralizing antibodies (A,B) early after vaccination, (C,D) late after vaccination, (E,F) 6 months after infection, and (G,H) early after booster vaccination.

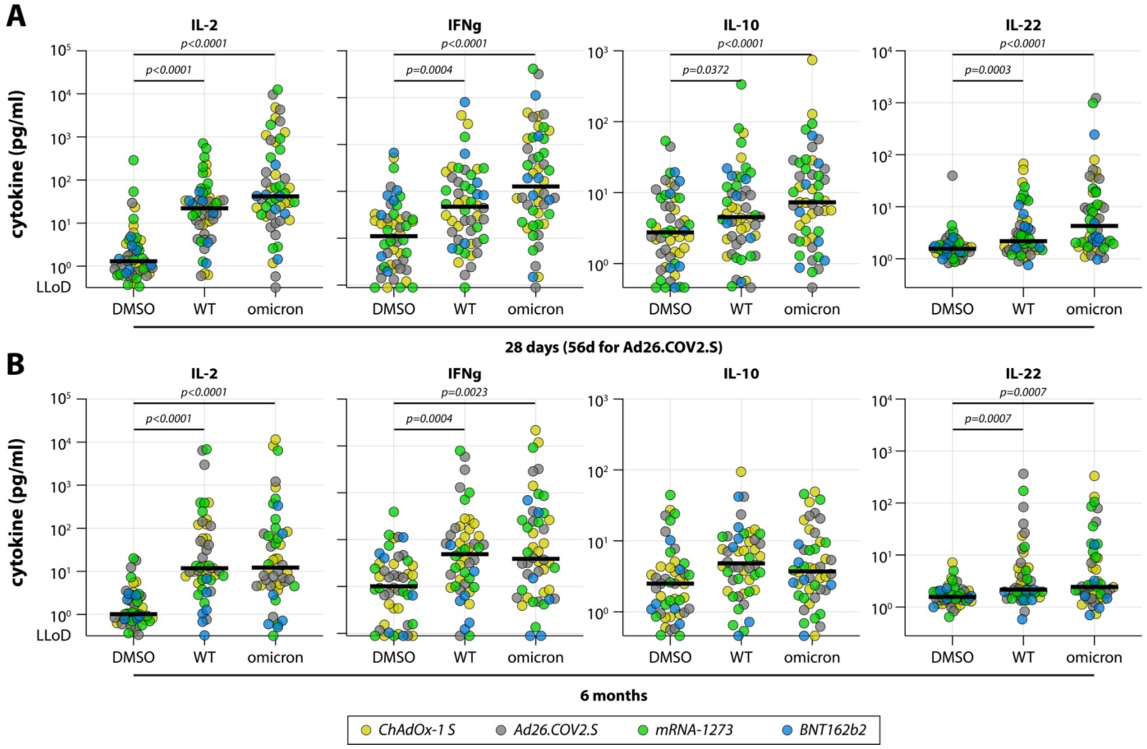

Figure S3. SARS-CoV-2 variant-specific cytokine production. PBMC were stimulated with different overlapping peptide pools for 20 hours, followed by measurement of 13 cytokines in cell culture supernatant. Four cytokines were produced after peptide pool stimulation. (C, D) Production of IL-2, IFN-γ, IL-10 and IL-22 early (A) and late (B) after completion of the vaccination. Values indicate the concentration of the cytokine in pg/ml supernatant, bars indicate the means. Comparisons of VOC-specific responses within study groups were performed by Friedman test with multiple comparisons. LLoD = lower limit of detection, PBMC = peripheral blood mononuclear cells, IL = interleukin, IFN = interferon, DMSO = dimethylsulfoxide, WT = wildtype, d = days.

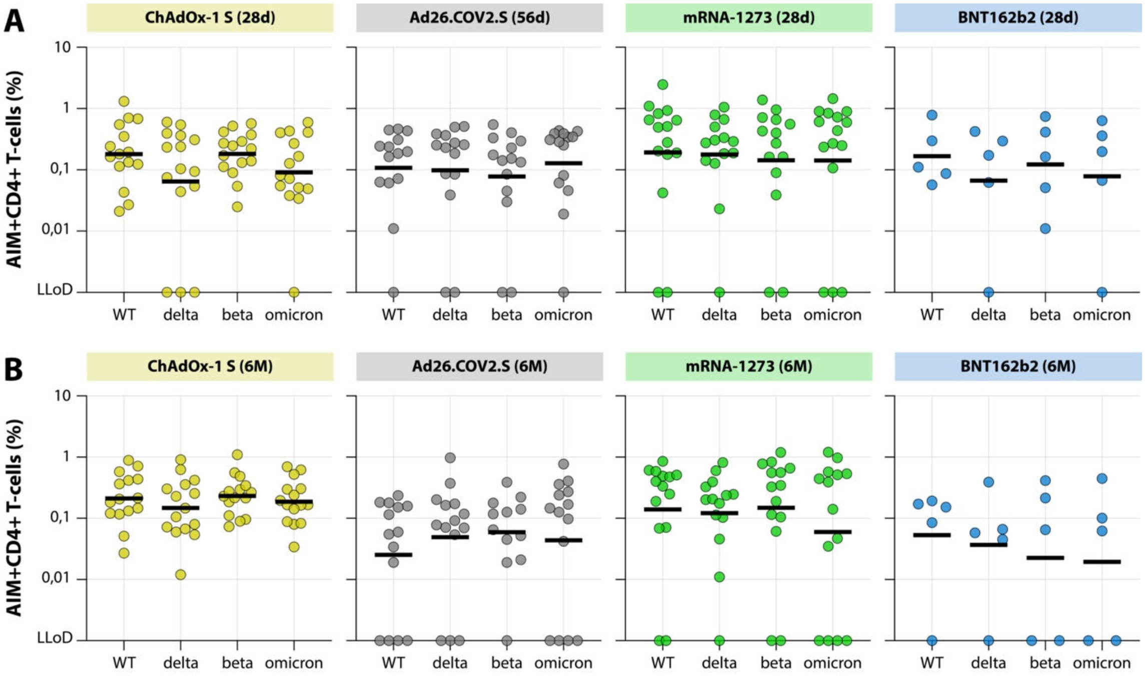

Figure S4. SARS-CoV-2 variant-specific CD4+ T-cell responses. (A, B) Upregulation of OX40 and CD137 early (A) and late (B) after completion of the vaccination regimen on CD4+ T-cells. Percentages indicate the percentage of AIM+ T-cells after subtraction of observed background in a DMSO stimulation, bars indicate the means. Comparisons of VOC-specific responses within study groups were performed by Friedman test with multiple comparisons, no significant differences were observed. The BNT162b2-vaccinated group was not included in statistics due to low sample size. LLoD = lower limit of detection, PBMC = peripheral blood mononuclear cells, AIM = activation-induced markers, d = days, M = months.

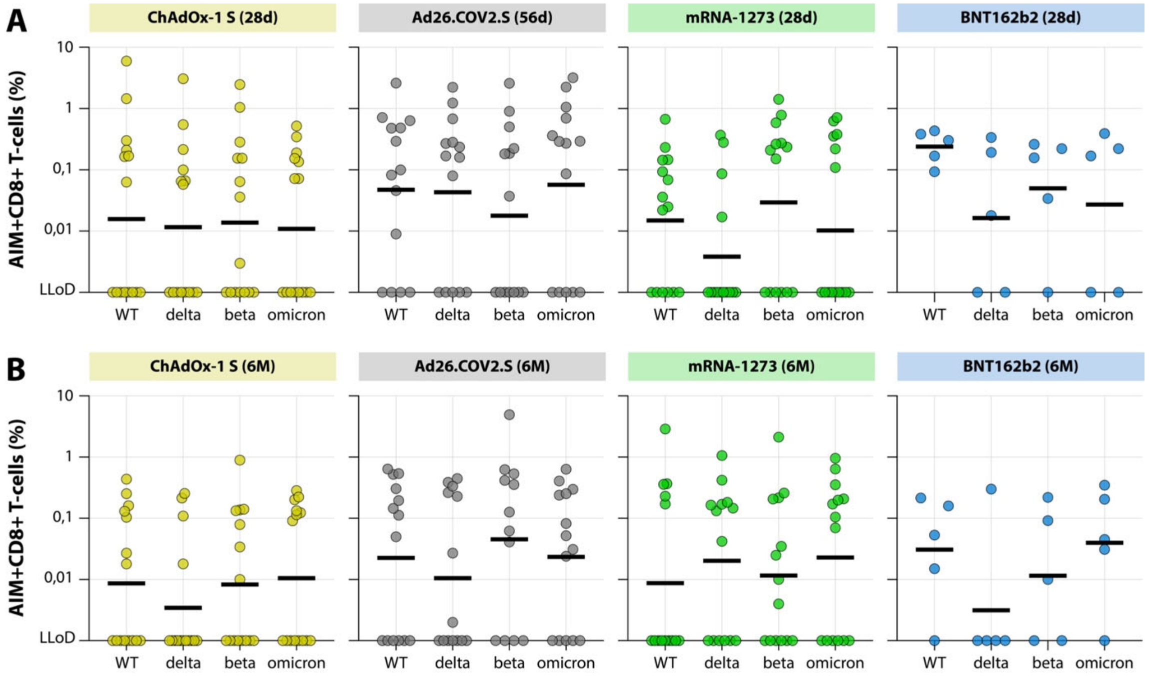

Figure S5. SARS-CoV-2 variant-specific CD8+ T-cell responses. (A, B) Upregulation of CD69 and CD137 early (A) and late (B) after completion of the vaccination regimen on CD8+ T-cells. Percentages indicate the percentage of AIM+ T-cells after subtraction of observed background in a DMSO stimulation, bars indicate the means. Comparisons of VOC-specific responses within study groups were performed by Friedman test with multiple comparisons, no significant differences were observed. The BNT162b2-vaccinated group was not included in statistics due to low sample size. LLoD = lower limit of detection, PBMC = peripheral blood mononuclear cells, AIM = activation-induced markers, d = days, M = months.

Supplemental Tables

Table S1. Cohort background. A total of 438 participants from three different trials (HCW, ConCOVID, SWITCH) were included in this study. * N = 9 participants of the HCW study were additionally analysed 14 days after the 3rd vaccination. § Ad26.COV2.S samples were analysed 56 days and 6 months after 1st vaccination. # T-cell responses were exclusively assessed in participants of the HCW study for vaccine regimen comparisons.

Study	Objective	Participants#	Timepoints
HCW	Vaccine regimen comparison	N = 400	28 days and 6 months post vaccination*§
ConCOVID	Convalescent patients	N = 23	6 months post infection
SWITCH	Heterologous vaccination	N = 15	28 days post 2nd vaccination
